# Personality and fatigue: meta-analysis of seven prospective studies

**DOI:** 10.1038/s41598-022-12707-2

**Published:** 2022-06-01

**Authors:** Yannick Stephan, Angelina R. Sutin, Martina Luchetti, Brice Canada, Antonio Terracciano

**Affiliations:** 1 Euromov, University of Montpellier, UFRSTAPS, 700, Avenue du Pic St Loup, 34090 Montpellier, France; 2 Department of Behavioral Sciences and Social Medicine, College of Medicine, Florida State University, Tallahassee, USA; 3 L-Vis, University Lyon 1, Villeurbanne, France; 4 Department of Geriatrics, College of Medicine, Florida State University, 1115 West Call Street, Tallahassee, FL 32306 USA

**Keywords:** Psychology, Signs and symptoms, Fatigue

## Abstract

The present study examined the cross-sectional and longitudinal associations between the five major personality traits and fatigue. Participants were adults aged 16–104 years old (N > 40,000 at baseline) from the Health and Retirement Study, the National Social Life, Health, and Aging Project, the Wisconsin Longitudinal Study graduate and sibling samples, the National Health and Aging Trends Survey, the Longitudinal Internet Studies for the Social Sciences and the English Longitudinal Study of Ageing. Personality traits, fatigue, demographic factors, and other covariates were assessed at baseline, and fatigue was assessed again 5–20 years later. Across all samples, higher neuroticism was related to a higher risk of concurrent (meta-analytic OR = 1.73, 95% CI 1.62–1.86) and incident (OR = 1.38, 95% CI 1.29–1.48) fatigue. Higher extraversion, openness, agreeableness, and conscientiousness were associated with a lower likelihood of concurrent (meta-analytic OR range 0.67–0.86) and incident (meta-analytic OR range 0.80–0.92) fatigue. Self-rated health and physical inactivity partially accounted for these associations. There was little evidence that age or gender moderated these associations. This study provides consistent evidence that personality is related to fatigue. Higher neuroticism and lower extraversion, openness, agreeableness, and conscientiousness are risk factors for fatigue.

## Introduction

Fatigue, which refers to a general feeling of tiredness, lack of energy and a loss of motivation, is a common complaint and a cause of consultation in primary care^1^. There is substantial evidence that fatigue is associated with poor outcomes in adulthood. For example, higher fatigue is indicative of the frailty syndrome^2^ and is associated with a higher risk of limitations in activities of daily living^3^. Furthermore, fatigue is associated with incident cardiovascular disease^4^ and higher risk of mortality^5,6^. Fatigue is a pluridetermined phenomenon, with determinants ranging from genetic^7^ to environmental^8^. The present study focused on the role of psychological factors by examining whether individuals’ personality traits are related to fatigue. The examination of the association between personality and fatigue can provide a better understanding of the extent to which fatigue is rooted in part in individuals’ psychological dispositions. In particular, the present study examines the association between personality and both concurrent and incident fatigue. Concurrent refers to the cross-sectional association between personality and fatigue when measured at the same time. The association between personality and incident fatigue refers to the relation between personality and new cases of fatigue over time among individuals who did not have fatigue at baseline.

Personality traits are relatively enduring patterns of thoughts, feelings and behaviors^9^. Existing models and research indicate that the personality traits defined by the Five-Factor Model (FFM)^10^ contribute to health across adulthood^11–13^. In particular, personality may be a valuable predictor of fatigue because it is associated with a range of health-related and behavioral factors that cause fatigue. Indeed, neuroticism (the propensity to experience negative emotions and distress) is a risk factor for poor mental health^14^ and other disorders and conditions like metabolic syndrome^15^, frailty^16^, and sleeping difficulties^17^. In turn, a common symptom of these disorders is fatigue and loss of energy^2,18–20^. Furthermore, high neuroticism is related to lower aerobic capacity and lower energy expenditure during a challenging physical activity^21^. This lower energy level may also contribute to higher overall fatigue. In contrast, high conscientiousness (a measure of self-discipline and organization), extraversion (the propensity to experience positive emotions and to be sociable), openness (the tendency to be imaginative and curious), and agreeableness (i.e., the propensity to be altruistic and empathetic) are associated with better physical and mental health outcomes, such as lower frailty, less sleeping difficulties, and fewer depressive symptoms^14,16,17^ which may manifest into lower fatigue. Furthermore, both conscientiousness and extraversion are associated with lower allostatic load^22^, which is related to lower fatigue^23^. Higher conscientiousness, extraversion, and openness are also associated with greater aerobic capacity and energy expenditure^21^ and lower fatigability in a physical task^24^, which may translate into lower overall fatigue. Personality traits are also associated with behaviors that may have implications for fatigue. For example, higher neuroticism is related to physical inactivity and sedentary behaviors, whereas higher extraversion, openness, agreeableness, and conscientiousness are associated with a physically active lifestyle^25^. In turn, physical activity is associated with a reduced likelihood of low energy and fatigue^26^.

Consistent with these assumptions, there is evidence that personality predicts fatigue. Higher neuroticism has been found to relate to higher fatigue in the general population and in clinical samples^27–34^. Compared to neuroticism, findings for the other traits are less consistent. Higher extraversion has been associated with lower fatigue in some studies^28,32,35,36^, but not in others^30–33^. Some studies found a relationship between higher conscientiousness and lower fatigue^27,28,31^, whereas other studies did not report any association^30,32^. High agreeableness has been associated with lower fatigue in one study^32^, to higher fatigue in another study^33^, and unrelated to fatigue in other studies^30,31^. Likewise, no consistent association has been found between openness and fatigue^30–33^. Most research on the association between personality and fatigue has focused on clinical^37–40^ and small samples^27–30,32^, which may explain some inconsistent findings across studies. No large-scale study on the association between personality and fatigue has been conducted yet. Furthermore, most studies have been cross-sectional, and little is known about the extent to which personality predicts incident fatigue using long-term prospective data.

Based upon seven longitudinal samples of adults, the present study examined the association between personality and fatigue across adulthood. In line with past research^28–34^, it was hypothesized that higher neuroticism would be related to higher likelihood of concurrent fatigue. Given that a majority of research has found a link between higher extraversion and conscientiousness and lower fatigue^27,28,31,32,35,36^, these two traits were expected to be related to lower concurrent fatigue. Similarly, it was expected that higher neuroticism would be related to higher probability of incident fatigue over time, whereas higher extraversion and conscientiousness would be related to lower likelihood of incident fatigue. Since most research does not report a significant association between openness and agreeableness and fatigue^30–33^, no associations with these two traits were expected. Furthermore, health-related factors and physical activity have been associated with both personality^14,16,17,25^ and fatigue^2,18–20,26^. Therefore, additional analyses examined whether the link between personality and fatigue was accounted for by self-rated health and physical inactivity. Exploratory analysis also tested the moderating role of demographic factors in the relation between personality and fatigue to examine whether this association varies depending on age, sex, education and race.

## Method

### Participants

This study was not pre-registered. Participants were from the Health and Retirement Study (HRS), the National Social Life, Health, and Aging Project (NSHAP), the Wisconsin Longitudinal Study graduate (WLSG) and sibling (WLSS) samples, the National Health and Aging Trends Survey (NHATS), the Longitudinal Internet Studies for the Social Sciences (LISS), and the English Longitudinal Study of Ageing (ELSA). In all cohorts, participants provided written informed consent, and all cohorts were conducted according to the principles expressed in the declaration of Helsinki. The present study was exempted from Institutional Review Board review because it used de-identified publicly available datasets. Descriptive statistics for the seven samples are presented in Table 1.

**Table 1:** Baseline characteristics of the samples.

Variables	HRS		NHATS		NSHAP		WLSG		WLSS		LISS		ELSA	
	M/%	SD	M/%	SD	M/%	SD	M/%	SD	M/%	SD	M/%	SD	M/%	SD
Age (years)	68.51	9.87	79.47	7.53	72.34	7.05	53.21	0.62	53.51	7.38	45.98	15.66	66.08	8.68
Sex (% women)	59%	–	59%	–	53%	–	54%	–	53%	–	54%	–	55%	–
Race (% white)	85%	–	72%	–	80%	–	100%	–	100%	–	100%	–	98%	–
Education	12.83	2.94	5.20	2.26	2.80	1.00	13.71	2.31	13.79	2.54	3.83	1.67	4.17	2.23
Neuroticism	2.04	0.61	2.22	0.86	1.40	0.59	3.21	0.98	3.22	0.95	2.58	0.68	2.10	0.59
Extraversion	3.20	0.55	3.13	0.76	2.20	0.56	3.83	0.89	3.76	0.90	3.29	0.63	3.15	0.56
Openness	2.95	0.55	2.81	0.84	1.91	0.65	3.65	0.80	3.61	0.75	3.51	0.50	2.88	0.55
Agreeableness	3.53	0.47	3.57	0.56	2.46	0.51	4.75	0.73	4.69	0.74	3.91	0.49	3.51	0.48
Conscientiousness	3.36	0.48	3.20	0.75	2.35	0.55	4.87	0.68	4.78	0.71	3.73	0.52	3.30	0.49
Fatigue (%)	32%	–	47%	–	52%	–	60%	–	60%	–	31%	–	26%	–
Incident fatigue (%)^a^	20%		37%		38%		39%		40%		19%		17%	

HRS: N = 12,364; NHATS: N = 2760; NSHAP: N = 2062; WLSG: N = 6590; WLSS: N = 3356; LISS: N = 5796; ELSA: N = 8078.

^a^Individuals who reported fatigue at baseline were excluded.

The HRS is a nationally representative longitudinal study of Americans older than 50 years and their spouse. Half of the sample provided demographic factors, personality, and fatigue data in 2006, and the other half provided these data in 2008. A baseline sample of 12,364 participants aged from 50 to 104 years old (59% women, mean age = 68.51, SD = 9.87) was obtained by combining both waves. Of this sample, follow-up data were obtained from 6,120 individuals in the 2018 wave. HRS data is publicly available at http://hrsonline.isr.umich.edu/. The HRS was approved by the University of Michigan Institutional Review Board (IRB).

The NSHAP is a longitudinal, population-based study of health and social factors among older community-dwelling Americans. Baseline data on personality traits, demographic factors, and fatigue were obtained in 2010–2011 (Wave 2) from 2062 individuals aged from 62 to 90 years (53% female, Mean age: 72.34, SD: 7.05). Follow-up data were obtained in 2015–2016 (Wave 3) from 1519 individuals. Information about NSHAP data can be found at: https://www.norc.org/Research/Projects/Pages/national-social-life-health-and-aging-project.aspx. The NSHAP was approved by the National Opinion Research Center and the University of Chicago IRBs.

The WLS is a long-term study of a random sample of 10,317 men and women who graduated from Wisconsin high schools in 1957, which is broadly representative of white, non-Hispanic American men and women who graduated from high school in Wisconsin at the time (WLSG). Baseline demographic, personality, and fatigue data were obtained in 1992–1993 from a total of 6,590 participants aged from 51 to 56 years old (54% women, mean age = 53.21, SD = 0.62). Of this sample, 4233 participants provided follow-up fatigue data in 2011. Selected siblings of some of the graduates were also included in the WLS (WLSS). A total of 3356 participants aged from 29 to 79 years old (53% women, mean age = 53.51, SD = 7.38) provided complete baseline data in 1993–1994. From this sample, follow-up fatigue data were obtained from 1948 participants in 2011. A public use file of data is available at http://www.ssc.wisc.edu/wlsresearch/data/. The WLS received approval from the Health Sciences IRB at University of Wisconsin–Madison.

The LISS is a representative longitudinal sample of the Dutch population. Complete baseline personality, demographic, and fatigue data were obtained in 2007 from a total of 5,796 participants aged from 16 to 94 years old (54% women, mean age = 45.98, SD = 15.66). Within this sample, follow-up fatigue was obtained in 2020 from 1,903 individuals. Information about the LISS panel can be found at: www.lissdata.nl. Ethical approval for the the LISS panel was given by the board of overseers.

ELSA is a representative cohort of men and women living in England aged 50 years and older. A sample of 8078 individuals aged from 50 to 89 years (55% women, mean age = 66.08, SD = 8.68) provided complete baseline demographic, personality, and fatigue data at wave 5 (2011). From this sample, a total of 5093 participants provided follow-up fatigue data at wave 9 (2018). Information about ELSA can be found at: https://www.elsa-project.ac.uk/. ELSA was approved by the National Research and Ethics Committee of the UK National Health Service.

The NHATS is a nationally representative longitudinal study of Medicare enrollees aged 65 years and older. Personality was first assessed in 2013 for one-third of the sample, and in 2014 for a second third. Both waves were combined. A sample of 2760 participants aged from 67 to 89 years (59% women, mean age = 79.47, SD = 7.53) provided complete data on demographic factors, personality, and self-rated health at baseline. Follow-up fatigue data was obtained in 2020 from 1,270 individuals. NHATS data are available at: https://nhats.org/. The NHATS was approved by the Johns Hopkins Bloomberg School of Public Health Institutional Review Board.

### Measures

#### Personality

Participants completed the Midlife Development Inventory (MIDI)^41^ in the HRS, ELSA, NSHAP, and NHATS. A 26 version of the MIDI was used in the HRS and ELSA, a 21-item version was used in the NSHAP, and a 10 item version was used in the NHATS. They were asked to indicate on a scale ranging from 1 (*not at all*) to 4 (*a lot*) how well adjectives assessing the five traits described themselves. Example adjectives are: worrying (neuroticism), talkative (extraversion), creative (openness), caring (agreeableness), and organized (conscientiousness). The 50 item International Personality Item Pool (IPIP)^42^ was used in the LISS. Participants were asked to rate each item on a scale from 1 (*Very inaccurate*) to 5 (*Very accurate*). Example items are: “get stressed out easily” (neuroticism), “start conversations” (extraversion), “have a vivid imagination” (openness), “sympathize with others’ feelings” (agreeableness), and “follow a schedule” (conscientiousness). In both the WLSG and the WLSS, a 29-item version of the Big Five Inventory^43^ was used. A 6-point scale ranging from 1 (*disagree strongly*) to 6 (*agree strongly*) was used to rate the extent to which participants agreed with descriptive statements such as: “To what extent do you agree that you see yourself as someone who worries a lot?” (neuroticism), “To what extent do you agree that you see yourself as someone who is outgoing and sociable?” (extraversion), “To what extent do you agree that you see yourself as someone who has an active imagination?” (openness), “To what extent do you agree that you see yourself as someone who is considerate to almost everyone?” (agreeableness) and “To what extent do you agree that you see yourself as someone who does things efficiently?” (conscientiousness). Cronbach alphas ranged from 0.67 to 0.88 for neuroticism, from 0.75 to 0.86 for extraversion, from 0.57 to 0.79 for openness, from 0.68 to 0.80 for agreeableness, and from 0.64 to 0.77 for conscientiousness.

#### Fatigue

Building on prior studies^44^, fatigue was assessed using two questions from the Center for Epidemiologic Studies-Depression (CES-D) scale^45,46^ in the WLS, the HRS, ELSA, and the NSHAP. In the WLS, the two questions asked how many days during the last week individuals felt that everything they did was an effort and whether they could not get going. Answers ranged from 0 for none to 7 for every day in the past week. These responses were recoded to 0 for none and 1 for at least 1 day in the past week. The same items were used in the HRS and ELSA, except that individuals were asked whether or not they had experienced these symptoms much of the time in the past week, using a yes/no format. In the NSHAP, individuals were asked to indicate how often during the past week they experienced these symptoms using a scale ranging from 1 for rarely or none of the time to 4 for most of the time. Answers were recoded to 0 for none and 1 for at least some of the time. Fatigue was defined as a positive response to at least one of the two questions. In the NHATS, participants were asked whether they had low energy or were easily exhausted during the last month using a yes/no format. In the LISS, participants were asked to report whether they regularly suffered from fatigue using a yes/no format.

#### Covariates

Age (in years), sex (coded as 1 for male and 0 for female), and education were included as covariates. Race was included in the HRS, ELSA, and NHATS (coded as 1 for white and 0 for other). Years of education were reported in the WLSG, WLSS, and HRS. Education was measured on a scale ranging from 1 (No schooling completed) to 9 (Master’s, professional or doctoral degree) in the NHATS, from 1 (No qualification) to 7 (NVQ4/NVQ5/Degree or equivalent) in the ELSA, from 0 (not yet completed any education) to 7 (other) in the LISS, and from 1 “less than high school” to 4 “bachelors or more” in the NSHAP. These demographic factors were included as covariates because of their association with fatigue^47^.

Self-rated health and physical inactivity were included as covariates in additional analyses, given their relationship with fatigue in past research^48^. Self-rated health was also included because it is is a reliable, inclusive measure of overall health status^49^. It was assessed using a single-item measure in the seven samples. For example, HRS used the following item: “Would you say your health is excellent, very good, good, fair, or poor?” In the HRS, the ELSA, the NHATS, the NSHAP, and the LISS, a scale from 1 (poor) to 5 (excellent) was used, whereas participants rated their health on a scale from 1 (very poor) to 5 (excellent) in the WLSG and WLSS. In the HRS and ELSA, physical inactivity was the mean of two items asking how often individuals participated in vigorous and moderate physical activity on a scale from 1 (more than once a week) to 4 (hardly ever or never). In the NSHAP, participants were asked to report their frequency of vigorous physical activity on a scale ranging from 0 (never) to 5 (five or more times a week). In NHATS, individuals were asked to report whether they ever go walking for exercise (yes/no) and ever spent time on vigorous activities in the last month (yes/no). Responses to the two items were added. In the LISS, participants were asked to report on how many days during the last seven days they performed a strenuous and moderately intense physical activity. The maximum number of days of either type of activity was selected as the measure of physical activity. Finally, in the WLS samples, the mean of two items asking how often individuals participated in light and vigorous physical activity on a scale from 1 (three or more times per week) to 4 (less than once per month) was computed.

### Data analysis

All analyses were conducted for each trait and in each sample separately and the results combined with random-effect meta-analyses using the Comprehensive Meta-Analysis software (Version 2)^50^. The random-effects method was used based on the assumption that the underlying true effects may differ across samples. Heterogeneity was assessed using the I^2^ indicator^51^. Personality traits were z-scored (Mean = 0 and SD = 1) to report all personality effects as a 1 SD difference. In cross-sectional analyses, logistic regressions were conducted to test whether personality traits were related to the risk of concurrent fatigue at baseline. In longitudinal analyses, logistic regressions were conducted to test whether personality traits predict incident fatigue at follow-up among individuals who did not report fatigue at baseline (individuals who reported fatigue at baseline were excluded from this analysis). Both the cross-sectional and longitudinal analyses tested basic models that included age, sex, education, and race (available in the HRS, ELSA, NHATS, and NSHAP) as covariates. Self-rated health and physical inactivity were further included together as covariates in addition to demographic factors in follow-up cross-sectional and longitudinal analyses. The extent to which the associations between personality and fatigue were moderated by age and sex was examined. The interaction between each personality trait and either age or sex was tested to predict the probability of concurrent and incident fatigue.

## Results

Descriptive statistics are in Table 1. In line with the hypothesis, neuroticism was related to higher likelihood of concurrent fatigue (Table 2). This association was found in the seven samples and the meta-analysis. For every standard deviation increase in neuroticism, there was a 70% increase of the probability of fatigue. As hypothesized, higher extraversion and higher conscientiousness were related to a lower risk of concurrent fatigue, an effect that was similar across all seven samples (Table 2): a one SD higher extraversion and conscientiousness were related to about 35% and 50% reduced probability of fatigue. Unexpectedly, higher openness and agreeableness were also related to lower concurrent fatigue in the meta-analysis (Table 2). The associations for the traits were attenuated by about 5% (Agreeableness) to 11% (Extraversion) but remained significant when self-rated health and physical activity were included as covariates, except for openness (see “Supplementary Material S1”).

**Table 2:** Summary of logistic regression analysis predicting baseline fatigue from baseline personality traits.

	HRS^a^	NHATS^a^	NSHAP^a^	WLSG^b^	WLSS^b^	LISS^b^	ELSA^a^	Pooled odds ratio	Heterogeneity I^2^
Neuroticism	1.81*** (1.73–1.89)	1.56*** (1.44–1.69)	1.61*** (1.46–1.77)	1.60*** (1.52–1.69)	1.66*** (1.54–1.79)	1.93*** (1.82–2.06)	1.96*** (1.86–2.08)	1.73*** (1.62–1.86)	88.26
Extraversion	0.67*** (0.65–0.70)	0.85*** (0.79–0.92)	0.65*** (0.59–0.71)	0.76*** (0.72–0.79)	0.82*** (0.77–0.88)	0.86*** (0.82–0.91)	0.54*** (0.51–0.57)	0.73*** (0.64–0.83)	96.99
Openness	0.79*** (0.76–0.83)	0.95 (0.88–1.03)	0.85*** (0.78–0.93)	0.83*** (0.79–0.88)	0.88*** (0.81–0.95)	1.04 (0.98–1.10)	0.69*** (0.66–0.73)	0.86** (0.77–0.95)	94.95
Agreeableness	0.84*** (0.81–0.88)	0.95 (0.88–1.03)	0.85*** (0.77–0.93)	0.75*** (0.71–0.79)	0.73*** (0.68–0.78)	1.05 (0.99–1.11)	0.83*** (0.79–0.87)	0.85*** (0.78–0.93)	93.60
Conscientiousness	0.67*** (0.64–0.70)	0.76*** (0.70–0.82)	0.66*** (0.60–0.73)	0.63*** (0.60–0.67)	0.60*** (0.56–0.65)	0.83*** (0.78–0.88)	0.60*** (0.57–0.63)	0.67*** (0.62–0.74)	93.39

HRS: N = 12,364; NHATS: N = 2760; NSHAP: N = 2062; WLSG: N = 6590; WLSS: N = 3356; LISS: N = 5796; ELSA: N = 8078.

^a^Adjusted for age, sex, education, and race.

^b^Adjusted for age, sex, and education.

*p < 0.05, **p < 0.01, ***p < 0.001.

There was little evidence that age or sex moderated the association between personality and concurrent fatigue across the seven samples (see “Supplementary Material S1”).

Consistent with cross-sectional results and the hypothesis, longitudinal analyses revealed that neuroticism was related to higher probability of incident fatigue among individuals without fatigue at baseline (Table 3). This relationship was observed in all samples (Table3): for every one standard deviation higher neuroticism, the risk of incident fatigue increased by around 35%. Conscientiousness was associated with lower risk of incident fatigue in 6 out of 7 samples and the meta-analysis (Table 3): a one SD higher conscientiousness was associated with around 20% lower likelihood of incident fatigue. While the results were not significant in all samples, the meta-analysis also indicated that extraversion, openness and agreeableness were related to lower probability of incident fatigue. However, there was some heterogeneity in the pattern of associations. A significant association between higher extraversion and openness and lower risk of fatigue at follow-up, for example, was found in three out of the seven samples, whereas the association between agreeableness and incident fatigue was found in two samples (Table 3). The overall pattern of associations was reduced by 1% (Agreeableness) to 5% (Conscientiousness) when self-rated health and physical activity were included in the analyses (“Supplementary Material S1”).

**Table 3:** Summary of logistic regression analysis predicting incident fatigue from baseline personality traits.

	HRS^a^	NHATS^a^	NSHAP^a^	WLSG^b^	WLSS^b^	LISS^b^	ELSA^a^	Pooled odds ratio	Heterogeneity I^2^
Neuroticism	1.37*** (1.27–1.47)	1.25** (1.07–1.46)	1.45*** (1.24–1.69)	1.22*** (1.11–1.34)	1.39*** (1.20–1.62)	1.61*** (1.40–1.85)	1.45*** (1.33–1.58)	1.38*** (1.29–1.48)	57.59
Extraversion	0.82*** (0.77–0.89)	1.05 (0.91–1.23)	0.89 (0.77–1.04)	0.95 (0.86–1.04)	0.77*** (0.67–0.89)	0.93 (0.81–1.06)	0.85*** (0.78–0.93)	0.89*** (0.83–0.95)	59.24
Openness	0.90** (0.84–0.97)	1.00 (0.86–1.16)	0.86 (0.74–1.00)	0.96 (0.86–1.06)	0.83* (0.71–0.97)	0.93 (0.80–1.07)	0.92* (0.84–1.00)	0.92*** (0.88–0.96)	0
Agreeableness	0.94 (0.87–1.01)	1.00 (0.91–1.10)	0.90 (0.77–1.05)	0.81*** (0.73–0.89)	0.77*** (0.66–0.90)	0.97 (0.84–1.12)	1.02 (0.93–1.11)	0.92* (0.85–0.99)	70.61
Conscientiousness	0.80*** (0.74–0.86)	0.86* (0.74–1.00)	0.86* (0.74–0.99)	0.80*** (0.73–0.89)	0.81** (0.70–0.93)	0.89 (0.77–1.02)	0.74*** (0.68–0.81)	0.80*** (0.77–0.84)	15.38

Note. HRS: N = 4630; NHATS: N = 749; NSHAP: N = 778; WLSG: N = 1739; WLSS: N = 796; LISS: N = 1359; ELSA: N = 3952.

^a^Adjusted for age, sex, education, and race.

^b^Adjusted for age, sex, and education.

*p < 0.05, **p < 0.01, ***p < 0.001.

## Discussion

The present study examined the association between personality traits and fatigue in seven large longitudinal samples of adults. As expected, the meta-analysis revealed that higher neuroticism was related to higher probability of concurrent and incident fatigue, whereas higher extraversion and conscientiousness were associated with a lower likelihood of fatigue both concurrently and over time. Unexpectedly, the meta-analyses revealed that higher openness and agreeableness were related to a lower risk of concurrent and incident fatigue. The associations were attenuated but remained significant when both self-rated health and physical inactivity were included as covariates. Furthermore, this pattern of associations was robust, with similar effects across cohorts that differed in terms of sample characteristics (e.g., ranging from a Medicare sample of older Americans to an internet sample with a broad age range in the Netherlands), across different measures of personality and fatigue, and with follow-ups ranging from about 5–20 years. Finally, this association was relatively independent of both chronological age and sex across the seven samples. To our knowledge, this study used the largest collection of samples to date to examine the association between personality and fatigue and found novel evidence for a prospective association between personality and incident fatigue.

As expected, higher neuroticism was related to a higher likelihood of concurrent and incident fatigue. This finding is consistent with existing cross-sectional evidence^28–34^, and extends past studies by showing that neuroticism is also related to higher risk of incident fatigue. It is likely that the basic tendencies associated with neuroticism, such as a higher propensity to experience distress and intense negative emotions, may translate into more fatigue. Furthermore, neuroticism is related to worse physical and mental health^11^ and health-risk behavior such as higher physical inactivity^25^, which are in turn linked to fatigue. Consistent with this assumption, self-rated health and physical inactivity partially accounted for the link between neuroticism and both concurrent and incident fatigue. However, the association between neuroticism and fatigue may operate through other biological and behavioral pathways. For example, higher neuroticism is related to metabolic syndrome^15^, lower energy expenditure during challenging physical tasks^21^, sleeping difficulties^17^, and persistent pain^52^, which are in turn linked to more fatigue^19,20^. Shared genetic factors may also partly explain the link between neuroticism and fatigue^7^.

As hypothesized, both extraversion and conscientiousness were related to lower probability of fatigue concurrently and over time. These results add to a mixed literature that has reported either positive or no associations between extraversion and conscientiousness and fatigue^27,28,31–33,35,36^. Furthermore, this pattern of association complements recent findings of an association between higher extraversion and conscientiousness and lower fatigability^24^. This study also contributes to existing knowledge by revealing that these traits are predictive of lower likelihood of incident fatigue over time. Many potential mechanisms may contribute to these associations. First, a core facet of extraversion is the propensity to be energetic and active, and core facets of conscientiousness are achievement-striving and industriousness. These basic tendencies may manifest in higher energy and lower tiredness. Second, both extraversion and conscientiousness may predict fatigue through indirect pathways. Higher extraversion and conscientiousness are related to better overall physical and mental health^11^, lower allostatic load^22^, a physically active lifestyle^25^, lower persistent pain^52^, lower stress reactivity^53^, and better sleep quality^17^, which may be conducive to lower fatigue. Accordingly, self-rated health and physical activity partly accounted for the associations between personality and fatigue.

Unexpectedly, higher openness and agreeableness were related to lower concurrent and incident fatigue, although the effect sizes were smaller and less consistent across samples compared to the effects observed for the other traits. This result adds to the mixed literature about the association between agreeableness and fatigue^30–33^ and between openness and fatigue^30–33^. This finding also complements a recent report of a relationship between higher openness and lower fatigability^24^. Openness and agreeableness are related to better health-related^54^ and behavioral^25^ profiles that may translate into lower fatigue. In addition, higher openness is related to lower inflammation^55^, and higher energy expenditure during challenging physical tasks^21^, which may contribute to a reduced likelihood of fatigue.

The present study contributes to existing models linking personality traits to health^12^. It provides consistent evidence that fatigue partly reflects enduring patterns of thoughts, feelings, and behaviors. Furthermore, fatigue could be an explanatory factor of the link between personality and a range of outcomes. For example, fatigue is predictive of a higher risk of limitations in independent activities of daily living (IADL)^3^ and mortality^5^. Higher neuroticism and lower conscientiousness are consistent predictors of incident IADL^56^ and mortality^57^. Therefore, it is likely that higher fatigue may be a manifestation of the risk of disability and mortality among individuals with high neuroticism and lower conscientiousness.

The present study has several strengths, including the examination of the association between personality and concurrent and incident fatigue in seven large longitudinal samples. However, this study also has several limitations. The overall design of the study precludes from drawing causal interpretations. Indeed, although there is theoretical support for a predictive role of personality for fatigue, fatigue may also predict personality change. In addition, the present study only examined the broad five personality domains defined by the five-factor model^10^. More research is needed using a facet-level analysis of the association between personality and fatigue. In addition, future research may test whether there are non linear relationships between personality and fatigue and whether this relationship varies based on objective health status. Except for LISS, the samples were limited to older adults and were predominantly white. Therefore, additional research should examine whether the results generalize to other age and ethnic groups. Future research should also test whether the link observed between personality and fatigue in large non-clinical samples generalizes to clinical samples. Finally, both personality and fatigue were assessed through self-reported measures, and the observed association could partly reflect common method variance. It is important to note, however, that similar associations have been found with objective measures of physical endurance^21^.

In sum, the present study found replicable concurrent and longitudinal associations between personality and fatigue. Higher neuroticism was consistently related to a higher likelihood of concurrent and incident fatigue, whereas higher extraversion and conscientiousness were associated with lower probability of fatigue, concurrently and over time. Although less consistently, higher openness and agreeableness were also related to lower concurrent and incident fatigue. These meta-analytic findings provide robust estimates of the association between the five major personality dispositions and feelings of fatigue. From a practical perspective, personality assessment may contribute to identification of individuals at risk of fatigue who may benefit from interventions to prevent or reduce fatigue. For example, individuals with higher neuroticism, lower extraversion and lower conscientiousness may be targeted by physical activity programs or cognitive behavioral therapy that could alleviate the experience of fatigue^26,58^. Furthermore, interventions could be directed toward changing personality traits^59^, that may reduce the risk of fatigue.

## Supplementary Information

Supplementary Information.

## Data Availability

The Health and Retirement Study (HRS) is sponsored by the National Institute on Aging (NIA-U01AG009740) and conducted by the University of Michigan. HRS data is publicly available at http://hrsonline.isr.umich.edu/. The Wisconsin Longitudinal Study (WLS) has been supported principally by the National Institute on Aging (AG-9775, AG-21079, AG-033285, and AG-041868), with additional support from the Vilas Estate Trust, the National Science Foundation, the Spencer Foundation, and the Graduate School of the University of Wisconsin-Madison. A public use file of data is available at http://www.ssc.wisc.edu/wlsresearch/data/. The Longitudinal Internet Studies for the Social Sciences (LISS) panel data were collected by CentERdata (Tilburg University, The Netherlands) through its MESS project funded by the Netherlands Organization for Scientific Research. More information about the LISS panel can be found at: www.lissdata.nl. Funding for the English Longitudinal Study of Ageing is provided by the National Institute of Aging [Grants 2RO1AG7644-01A1 and 2RO1AG017644] and a consortium of UK government departments coordinated by the Office for National Statistics. ELSA data are available from the UK Data Service (UKDS, https://www.ukdataservice.ac.uk/). The National Health, Social Life and Aging Project (NSHAP) is supported by the National Institutes of Health, including the National Institute on Aging, the Office of Women’s Health Research, the Office of AIDS Research, and the Office of Behavioral and Social Sciences Research (Grants R01 AG021487, R37 AG030481, R01 AG033903, R01 AG043538, and R01 AG048511). Information on how to access the NSHAP data can be found at: http://www.norc.org/Research/Projects/Pages/national-social-life-health-and-aging-project.aspx. The National Health and Aging Trends Study (NHATS) is sponsored by the National Institute on Aging (Grant number NIA U01AG032947) through a cooperative agreement with the Johns Hopkins Bloomberg School of Public Health. NHATS data are available for public download at: http://www.nhats.org.
