# Supplementary material for: Personality and fatigue: meta-analysis of seven prospective studies
Source: Sci Rep. 2022 Jun 1;12:9156. doi: 10.1038/s41598-022-12707-2 (PMC9160011; doi:10.1038/s41598-022-12707-2)
Supplement: Supplementary file 1 — Supplementary Information. [file 41598_2022_12707_MOESM1_ESM.docx]

**Supplementary Analyses**

Although higher neuroticism was related to higher likelihood of fatigue across all ages, this association was slightly stronger among younger individuals in ELSA (OR_Interaction_= .99, 95%CI: .98-.99, p<.001), HRS (OR _Interaction_ = .99, 95%CI: .99-1.00, p< .01) and WLSS (OR _Interaction_ = .99, 95%CI: .98-1.00, p<.05). Higher conscientiousness was more strongly related tossss lower risk of fatigue among younger individuals in the HRS (OR _Interaction_ = 1.01, 95% CI: 1.00-1.01, p<.01). Higher extraversion was more strongly related to lower fatigue among older individuals in the WLSS (OR _Interaction_ = .99, 95% CI : .98-1.00, p< .01) and the HRS (OR _Interaction_ = .99, 95% CI : .99-1.00, p< .05). Openness was related to a lower likelihood of fatigue among men in the WLSS (OR _Interaction_ = 1.17, 95%CI: 1.01-1.35, p< .05). In the LISS, higher neuroticism was more strongly related to higher risk of fatigue among men (OR _Interaction_ = .76, 95%CI: 67-87, p<.001), and conscientiousness was more strongly associated with a lower likelihood of fatigue among men (OR _Interaction_ = .66, 95%CI: .63-70, p<.001).

Similar to the cross-sectional analyses, there was little evidence that age or gender moderated any of the associations (see supplementary material). In the WLSG, higher extraversion was related to lower likelihood of incident fatigue among younger individuals (OR _Interaction_= 1.22, 95%CI: 1.03-1.44, p< .05). The link between higher conscientiousness and lower risk of incident fatigue was more pronounced among younger individuals in ELSA (OR _Interaction_ = 1.01, 95%CI: 1.00-1.02, p<.05). Conscientiousness was related to lower likelihood of fatigue among women in the NSHAP (OR _Interaction_ = .68, 95%CI: .51-.92, p< .05). Openness was related to lower likelihood of incident fatigue among women in the HRS (OR _Interaction_ = 1.19, 95%CI: 1.02-1.39, p<.05).

Table S1

*Summary of Logistic Regression Analysis Predicting Baseline Fatigue from Baseline Personality Traits, Controlling for Self-Rated Health and Physical Activity*

HRS ^a^ NHATS ^a^ NSHAP^a^ WLSG ^b^ WLSS ^b^ LISS ^b^  ELSA^a^ Pooled Heterogeneity

Odds Ratio I^2^

| Neuroticism | 1.58***  (1.51-1.66) | | 1.44***  (1.32-1.57) | | 1.46***  (1.32-1.61) | 1.54***  (1.46-1.63) | | 1.61***  (1.49-1.74) | | 1.67***  (1.56-1.78) | | 1.77***  (1.66-1.88) | | 1.59***  (1.51-1.67) | | 73.52 | |  |
| --- | --- | --- | --- | --- | --- | --- | --- | --- | --- | --- | --- | --- | --- | --- | --- | --- | --- | --- |
| Extraversion | | 0.79***  (0.76-0.83) | | 0.94  (0.87-1.02) | 0.74***  (0.67-0.82) | | 0.78***  (0.74-0.82) | | 0.84***  (0.78-0.90) | | 0.92**  (0.87-0.98) | | 0.68***  (0.65-0.73) | | 0.81***  (0.75-0.88) | | 91.01 | |
| Openness | | 0.88***  (0.84-0.92) | | 1.04  (0.96-1.14) | 0.96  (0.87-1.06) | | 0.85***  (0.80-0.89) | | 0.89**  (0.82-0.96) | | 1.10**  (1.04-1.18) | | 0.78***  (0.74-0.83) | | 0.92  (0.84-1.01) | | 92.62 | |
| Agreeableness | | 0.90***  (0.86-0.94) | | 1.03  (0.95-1.12) | 0.92  (0.83-1.01) | | 0.77***  (0.73-0.81) | | 0.75***  (0.69-0.81) | | 1.08*  (1.01-1.15) | | 0.87***  (0.82-0.92) | | 0.89*  (0.81-0.98) | | 93.70 | |
| Conscientiousness | | 0.78***  (0.74-0.81) | | 0.83***  (0.76-0.91) | 0.75***  (0.68-0.83) | | 0.65***  (0.62-0.69) | | 0.62***  (0.57-0.66) | | 0.89***  (0.84-0.95) | | 0.73***  (0.69-0.77) | | 0.74***  (0.68-0.82) | | 93.21 | |

Note. HRS: N= 12112; NHATS: N= 2758; NSHAP: N= 2059; WLSG: N= 6,472; WLSS: N= 3,279; LISS: N= 5,790; ELSA: N= 8073

^a^ Adjusted for age, sex, education, race, self-rated health and physical activity

^b^ Adjusted for age, sex, education, self-rated health and physical activity

* *p* < .05, ** *p* < .01, *** *p* <.001

Table S2

*Summary of Logistic Regression Analysis Predicting Incident Fatigue from Baseline Personality Traits, Controlling for Self-Rated Health and Physical Activity*

HRS ^a^ NHATS ^a^ NSHAP^a^ WLSG ^b^ WLSS ^b^ LISS ^b^  ELSA^a^ Pooled Heterogeneity

Odds Ratio I^2^

| Neuroticism | 1.26***  (1.17-1.37) | | 1.22*  (1.04-1.42) | | 1.41***  (1.21-1.66) | 1.19***  (1.07-1.31) | | 1.35***  (1.16-1.58) | | 1.50***  (1.30-1.74) | | 1.40***  (1.28-1.53) | | 1.32***  (1.24-1.41) | | 49.78 | |  |
| --- | --- | --- | --- | --- | --- | --- | --- | --- | --- | --- | --- | --- | --- | --- | --- | --- | --- | --- |
| Extraversion | | 0.90**  (0.84-0.97) | | 1.08  (0.93-1.26) | 0.97  (0.83-1.13) | | 0.97  (0.88-1.07) | | 0.76***  (0.66-0.88) | | 0.93  (0.81-1.07) | | 0.93  (0.85-1.01) | | 0.93*  (0.87-0.99) | | 49.58 | |
| Openness | | 0.96  (0.89-1.04)  ) | | 1.03  (0.88-1.20) | 0.92  (0.79-1.08) | | 0.98  (0.89-1.09) | | 0.83*  (0.71-0.97) | | 0.95  (0.82-1.09) | | 0.95  (0.87-1.04) | | 0.95*  (0.91-0.99) | | 0 | |
| Agreeableness | | 0.97  (0.90-1.05) | | 1.03  (0.88-1.20) | 0.92  (0.79 -1.08) | | 0.83***  (0.75-0.91) | | 0.78**  (0.67-0.91) | | 0.98  (0.85-1.14) | | 1.02  (0.93-1.12) | | 0.93  (0.86-1.01) | | 65.88 | |
| Conscientiousness | | 0.88***  (0.82-0.95) | | 0.88  (0.75-1.03) | 0.91  (0.7-1.06) | | 0.81***  (0.74-0.90) | | 0.81**  (0.70-0.94) | | 0.91  (0.79-1.05) | | 0.78***  (0.71-0.84) | | 0.84***  (0.80-0.89) | | 27.26 | |

Note. HRS: N= 4523; NHATS: N= 748; NSHAP: N= 777; WLSG: N= 1703; WLSS: N= 783; LISS: N= 1358; ELSA: N= 3949

^a^ Adjusted for age, sex, education, race, self-rated health and physical activity

^b^ Adjusted for age, sex, education, self-rated health and physical activity

* *p* < .05, ** *p* < .01, *** *p* <.001
